# Risks of prostate cancer and mortality in the city of Sharjah, United Arab Emirates

**DOI:** 10.3389/fonc.2023.1180902

**Published:** 2023-05-23

**Authors:** Zainab Al Shareef, Rula Al-Shahrabi, Fatemeh Saheb Sharif-Askari, Younis Alshamsi, Abdulqadir Al Zarooni, Noura AlKhayyal, Sameh S. M. Soliman, Riyad Bendardaf, Rabih Halwani

**Affiliations:** ^1^Department of Basic Medical Sciences, College of Medicine, University of Sharjah, Sharjah, United Arab Emirates; ^2^Research Institute for Medical and Health Sciences, University of Sharjah, Sharjah, United Arab Emirates; ^3^Department of Pharmacy Practice and Pharmacotherapeutics, College of Pharmacy, University of Sharjah, Sharjah, United Arab Emirates; ^4^Urology Department, Al Qassimi Hospital, Sharjah, United Arab Emirates; ^5^Urology Department, Shaikh Khalifa General Hospital, Umm Al Quwain, United Arab Emirates; ^6^Oncology Unit, University Hospital of Sharjah, Sharjah, United Arab Emirates; ^7^Department of Medicinal Chemistry, College of Pharmacy, University of Sharjah, Sharjah, United Arab Emirates; ^8^Department of Clinical Sciences, College of Medicine, University of Sharjah, Sharjah, United Arab Emirates

**Keywords:** benign prostatic hyperplasia, prostate cancer, PSAD, Gleason score, risk factors, all-cause mortality

## Abstract

**Background:**

Prostatic hyperplasia (BPH) and prostate cancer (PCa) are common age-related diseases in men. According to World Health Organization (WHO), PCa is the second most common cancer among Emirati men. This study aimed to identify the risk factors associated with PCa and mortality in a cohort diagnosed with PCa between 2012 and 2021 in Sharjah, United Arab Emirates (UAE).

**Methods:**

The data collected in this retrospective case-control study included patient demographics and comorbidities, as well as PCa markers such as prostate-specific antigen (PSA), prostate volume, prostate-specific antigen density (PSAD), and Gleason scores. Risk factors for PCa were assessed using multivariate logistic regression analysis, and factors associated with all-cause mortality in PCa patients were evaluated using Cox-proportional hazard analysis.

**Results:**

Of the 192 cases analyzed in this study, 88 were diagnosed with PCa and 104 were diagnosed with BPH. Regarding risk factors for PCa, a higher risk of PCa was associated with age 65 or older (OR=2.76, 95% confidence interval (CI): 1.04-7.30; P=0.038) and serum PSAD greater than 0.1 ng/mL^2^ (OR=3.48, 95% CI:1.66-7.32; P=0.001), whereas being of UAE nationals (OR=0.40, 95% CI:0.18-0.88; P=0.029) were associated with lower risk of PCa, after adjusting for patient demographics and comorbidities. Moreover, regarding cancer markers, higher serum PSA level (P=0.003) and smaller prostate volume (P=0.028) were associated with a higher risk of PCa, after adjusting with patients’ age and BMI. Additionally, a high-grade Gleason score was associated with an increased risk of all-cause mortality after adjusting for patient’s age and BMI (hazard ratio, aHR= 2.3, 95% CI:1.3-4.1; P= 0.016).

**Conclusion:**

This study found that age 65 or older and serum PSAD greater than 0.1 ng/mL^2^ are risk factors for PCa, while UAE nationality is associated with a lower risk. PSAD may be a better screening marker for PCa compared to traditional markers such as PSA and prostate volume.

## Introduction

Prostate diseases are common in men particularly after age 50 (1, 2). The walnut-size prostate secrets semen fluid locates below the bladder and surrounding the urethra (3). The enlargement of the prostate in benign prostatic hyperplasia (BPH) causes urinary tract-related symptoms such as weak urination, difficulty in emptying the bladder, urine dripping, nocturia and less likely haematuria and frequent urinary tract infection (UTI) (4). Bladder and kidney damage are complications related to the delayed treatment of PBH (5). Besides PBH, prostate cancer (PCa) is a common disease of the ageing prostate. PCa is the fifth leading cause of death in men and the second most common disease worldwide with an estimated 26,730 deaths in the USA (6). Although PCa is a silent killer in men because it’s asymptomatic in most patients (7), the possibility of recovery is very high when diagnosed at early stages (7). In the United Arab Emirates (UAE), there is a lack of prostate disease epidemiology and associated risk factors. Notably, according to WHO cancer statistics 2020, PCa is the second commonest cancer among Emirati males (8). Ethnicity is one of the disease risk factors; PCa is the highest among black Africans compared to Asians and Europeans (9). PBH is mostly diagnosed in black African and Latino patients (10). Reporting the incidence of prostate disease in relation to possible risk factors such as age, race, diet, comorbid diseases, and medication intake is essential to increase the awareness and the rate of PCa screening within men population (11). Assessing the level of prostate-specific antigen (PSA) is currently used in PCa screening (12). However, it’s a less specific biomarker that shows high false positive cases (healthy men diagnosed false with PCa) (12, 13). Furthermore, PSA can be positive with other non-cancer pathologies such as PBH and prostatitis (12, 13). Importantly, the effectiveness of screening tests and the risk factors of PCa in the gulf area and the UAE have not been investigated yet. This study aimed to investigate the risk factors and outcomes associated with PCa in a cohort of patients from Sharjah, UAE. Specifically, the study focused on the association between patient demographics, comorbidities, and cancer-specific markers such as serum PSA levels, prostate volume, PSAD, and Gleason score with the development and mortality of PCa. The findings of this study will provide valuable insights into the epidemiology of PCa in Sharjah and may inform clinical decision-making for the management and treatment of PCa patients in the region.

## Materials and methods

### Study design and population

In this retrospective case-control study of a total 192 cases, we examined the clinical characteristics of prostate diseases in men in the Sharjah population between January 2012 and December 2021. Data were collected for patients diagnosed with PCa or BPH at Al-Qassimi hospital and University Hospital Sharjah, the main healthcare providers in Sharjah, UAE. Sharjah accounts for 18.8% of the total UAE population. The retrieved data for each patient at diagnosis of BPH or PCa were as follows; basic demographics such as age, ethnicity, body mass index (BMI) and patient comorbidities including diabetes, hypertension, cardiovascular diseases, hyperlipidemia, presence of urinary tract infections (UTI), and chronic kidney diseases (CKD). Prostate cancer markers such as serum PSA, prostate volume, PSAD, and Gleason score were also recorded.

### Ethical approval

This study was approved by the University of Sharjah ethical committee under reference number: REC-20-03-23-01, the university hospital of Sharjah research committee Ref. No.: UHS-HERC-085-10012022 and the UAE Ministry of Health and Prevention (MOHAP), date of Amendment Request: 31/01/2022. Ethical approval code number: MOHAP/DXB-REC/JJA/No.75/2020. The informed patient consent form was waived by the research committee due to the retrospective study design.

### Statistical analysis

Categorical variables were presented by frequencies and percentages, while continuous data were presented as median. In the univariate analysis, the association between categorical variables was tested using the Chi-square test, while continuous variables were analyzed using Man-Whitney test depending on the skewness of data. In the multivariate analysis, factors contributing to PCa were identified using logistic regression analysis, and Cox proportional analysis was used to identify factors associated with mortality. To avoid strong correlations between the variables, all selected variables in the model were tested for multicollinearity. The variance inflation factors and the standard error magnitude were evaluated to determine whether collinearity existed. Multiple imputation techniques were used to impute prostate volume by creating 250 imputations. The pooled estimate was used for analysis. The Statistical Package for Social Sciences (SPSS) version 26 (IBM Corp, New York), R software (version 3.6.1), and PRISM software (version 8) was used to perform all the analyses. Significance was set at *P* value < 0.05.

### Hematoxylin and eosin staining

In microscope slides, 5-micron sections were cut from each block and fixed for 30 min in a hot plate at 60 °C. Sections were Deparaffinized with two xylene changes for 5 min each. Following that, sections were immersed in a series of graded alcohols, 100% ethanol for 10 min, 95% ethanol for 3 min, 70% ethanol for 2 min, and distilled water for 1 min. Slides were stained with Gill’s hematoxylin for 8 min and then rinsed under tap water to blue the nucleus. Cytoplasm staining was then performed by dipping the slides in 1% alcoholic eosin for 3 min. slides were dehydrated with 95% alcohol and two changes of 100% alcohol for 3 and 5 min, respectively. Two changes of xylene were used to extract the alcohol. One or two drops of mounting medium were added to the slide and covered with a coverslip. Slides were left to dry before microscopic examination (14).

## Results

### Patient characteristics

There were 192 cases in this study, 88 of which were PCa, and 104 of which were BPH, diagnosed between 2012 and 2021 in Sharjah. The demographics and clinical characteristics of patients are presented in **Table 1**. There was no difference between age of patients with PCa and BPH at diagnosis (median age of 71 and 72 years, respectively; P=0.513). The two groups did not differ in weight (BMI ≥ 25 kg/m^2^). PCa was more prevalent among non-Emiratis than Emiratis (68% versus 43%, respectively; P=0.001), and BPH was more prevalent among Emiratis.

**Table 1:** Patient characteristics associated with PCa or BPH.

Variables	PCa (n=88)	BPH (n=104)	p-value
Demographics			
Age, median (range) year	71 (67-77)	72 (65-79)	0.513
Emirates nationality, (%)	38 (43)	71 (68)	0.001
Comorbidities, (%)			
Diabetes mellitus	46 (52)	49 (47)	0.476
Hypertension	38 (43)	49 (47)	0.585
Cardiovascular diseases	16 (18)	11 (11)	0.131
Overweight (BMI ≥ 25 kg/m^2^)	66 (75)	64 (61)	0.063
Hyperlipidemia	29 (33)	42 (40)	0.288
Urinary tract infection	23 (26)	64 (61)	<0.001
Chronic kidney disease	5 (6)	15 (14)	0.048
Clinical pathological features			
PSA, median (range), ng/mL	11 (5-23)	5 (4-11)	<0.001
*Prostate volume, median (range) mL	54 (40-80)	70 (47-100)	0.032
PSAD, median (range), ng/mL^2^	0.20 (0.08-0.51)	0.08 (0.05-0.16)	0.004
Gleason score, (%)			
Low grade; Gleason score ≤6	25 (28)	–	
Moderate grade; Gleason score 7	28 (32)	–	
Gleason score 7 (3+4)	19 (22)	–	
Gleason score 7 (4+3)	9 (10)	–	
High grades; Gleason score 8-10	35 (40)	–	
Clinical outcome			
**Death**	15		

BMI, body mass index; BPH, Benign prostate hyperplasia; PCa, Prostate cancer; PSA, prostate-specific antigen; PSAD, prostate-specific antigen density.

*The prostate volume of 23 patients with PCa or BPH was missing (the total sample size; n=169).

Regarding comorbidities, both groups did not differ in the prevalence of diabetes mellitus (DM), hypertension, cardiovascular diseases, and hyperlipidemia. The prevalence of urinary tract infections was higher in BPH patients than in PCa patients (61% *vs.* 26%, respectively; P<0.001). A higher prevalence of chronic kidney disease was also observed in BPH patients than in PCa patients (14% *vs.* 6%, respectively; P=048).

According to cancer markers, serum PSA level and, subsequently PSAD level were significantly higher among PCa patients than BPH patients (PSA; 11 ng/ml *vs.* 5 ng/ml, respectively; P<0.001, and PSAD; 0.2 ng/ml^2^ *vs.* 0.08 ng/ml^2^, respectively; P=0.004). However, the prostate volume was larger among BPH patients than PCa patients (70 mL *vs.* 54 mL, respectively; P=0.032). Additionally, the Gleason scores of two-thirds of PCa patients were moderate (32%) to high (40%).

### Risk factors associated with PCa

**Figure 1:**
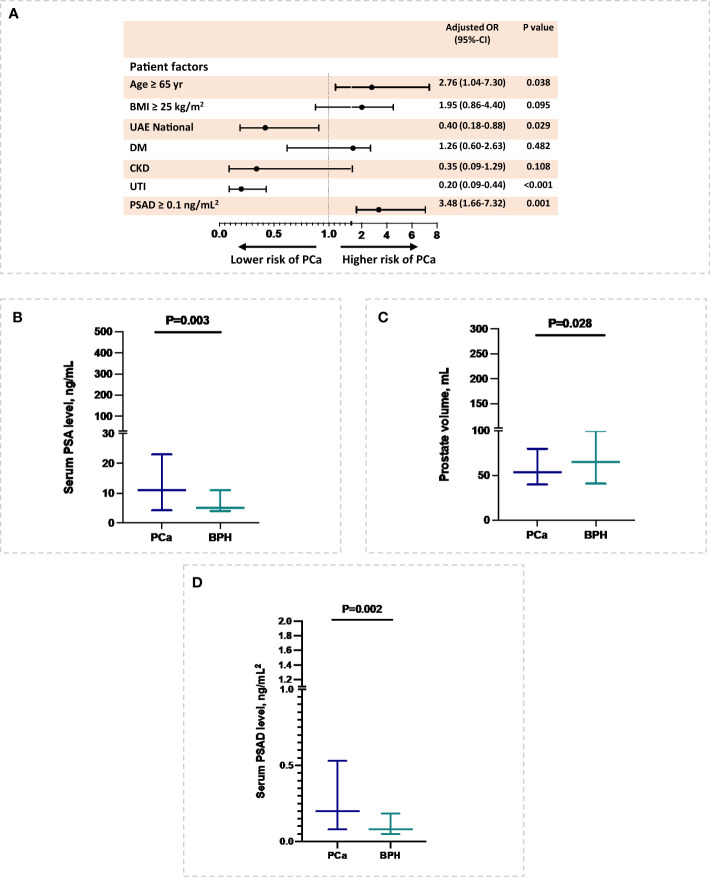
Patient’s risk factors of PCa. **(A)** Patient’s demographic and comorbidities associated with PCa. **(B-D)** Serum PSA level, prostate volume, and serum PSAD level at diagnosis in relation to PCa. The association of prostate volume with PCa was inferred with multiple imputations.

In this study, a higher risk of PCa was associated with age 65 or older (OR=2.76, 95% confidence interval (CI): 1.04-7.30; P=0.038) and serum PSAD greater than 0.1 ng/mL^2^ (OR=3.48, 95% CI:1.66-7.32; P=0.001), whereas being of UAE nationals (OR=0.40, 95% CI:0.18-0.88; P=0.029) were associated with lower risk of PCa, after adjusting for patient demographics, including age and nationality, comorbidities such as overweight, diabetes, CKD, and UTI, and serum PSAD level (**Figure 1A**). Moreover, in relation to cancer markers, a higher serum PSA level (P=0.003) and serum PSAD level (P=0.002), as well as a smaller prostate volume (P=0.028), were found to be associated with a higher risk of PCa after adjusting with patients’ age and BMI (**Figures 1B–D**).

### High-grade Gleason score was associated with all-cause mortality in PCa patients

**Figure 2:**
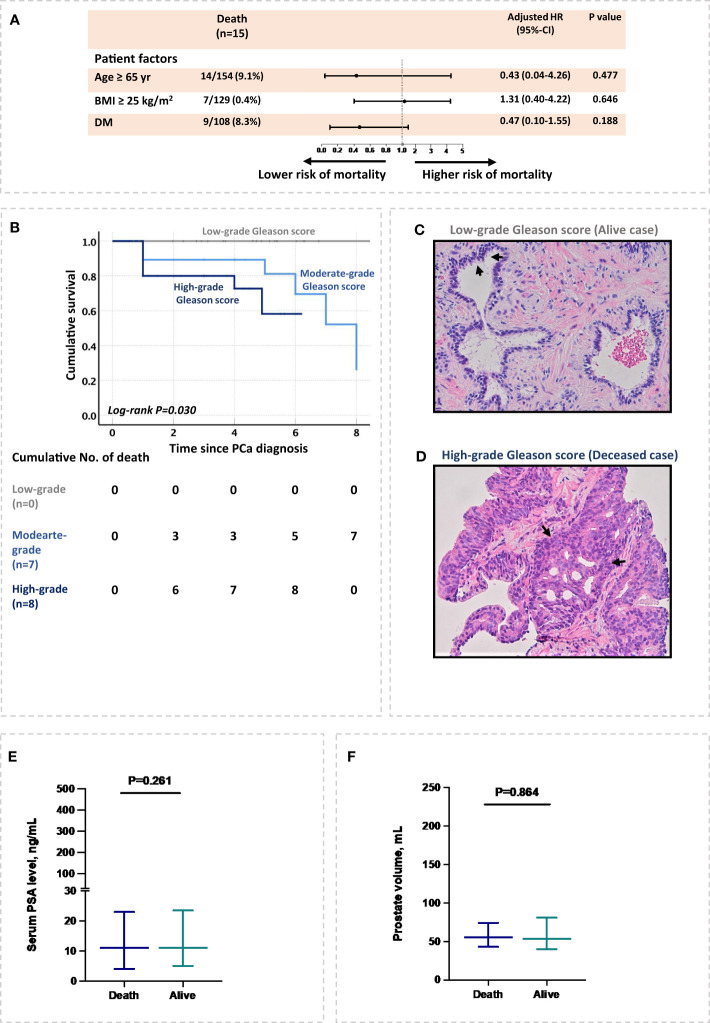
Risk factors associated with all-cause mortality in PCa patients. **(A)** Patient’s demographic and comorbidities associated with all-cause mortality. **(B)** High-grades Gleason scores associated with all-cause mortality. **(C, D)** patient’s biopsy stained with H&E. The figures represent two PCa patients, **(C)** an alive patient with low Gleason score, and **(D)** a deceased patient with a high-grade Gleason score. Both sections show adenocarcinoma and demonstrate nuclear and nucleolar enlargement with preservation of normal glandular architecture in the alive patient **(C)** compared to massive destruction of normal tissue architecture in deceased patient **(D)**. Remarkable acini growth infiltration and acini obliteration are presented in deceased patient **(D)**. **(E, F)** Serum PSA level and prostate volume at diagnosis in relation to all-cause mortality. The relation of prostate volume with PCa mortality was inferred with multiple imputations.

Furthermore, patient-specific factors such as age 65 or older, overweight, and diabetes mellitus were not associated with higher all-cause mortality in Cox proportional analysis (**Figure 2A**). Interestingly, among cancer markers, a high-grade Gleason score was associated with an increased risk of all-cause mortality after adjusting for patient’s age and BMI (hazard ratio, aHR= 2.3, 95% CI:1.3-4.1; P= 0.016) (**Figures 2B, C**). Additionally, cancer markers such as serum PSA level of prostate size were not associated with a higher risk of all-cause mortality in PCa cohort after adjusting for patient’s age and BMI (**Figures 2D–F**).

## Discussion

The findings of this study on PCa in Sharjah highlight several important factors that may impact the development and mortality of the disease. One of the most notable findings of this study is that older age and higher serum PSAD levels at diagnosis are strongly associated with an increased risk of PCa. Additionally, the study found that UTI comorbidity and a smaller prostate volume were associated with a lower risk of PCa. These findings suggest that certain comorbidities and physiological factors may play a protective role against the development of PCa.

Prostate cancer has become increasingly prevalent in numerous countries, including the United Arab Emirates (UAE). Nonetheless, there is limited understanding of the risk factors and outcomes linked to PCa in the Emirate of Sharjah, which is the second most populous emirate in the UAE with an estimated population of 1,324,473 (15). Our Study findings reveal that men in Sharjah are diagnosed with PCa at 67 years of age. Prostate cancer is frequently diagnosed in men above the age of 65, with nearly six out of ten cases worldwide falling in this age group (6, 16). The incidence of PCa peaks in Eastern Europe and Asia, where individuals 75 and older are affected (17). Six Arab countries, including Oman, Algeria, Syria, Tunisia, Lebanon, and Somalia, report a higher age-standardized incidence rate (ASIR) in men under 40 than the global ASIR (18). Kuwait and Lebanon have higher PCa ASIR among men aged 56-69 compared to the global ASIR (18). This could be due to a variety of factors, including underdiagnosis of the disease, overdiagnosis through screening tests, genetic predisposition, environmental factors, sedentary lifestyles, exposure to carcinogenic substances, or HPV infection (19). Underdiagnosis is defined as the failure to detect PCa, which may necessitate the initiation of screening tests at an earlier age of 45 rather than 50.

Prostate-specific antigen (PSA) has been widely used as a screening marker for PCa, despite its well-known limitations in terms of sensitivity and specificity. In our study, we found that PSA density (PSAD) was a better screening marker for PCa compared to PSA or prostate volume measured by transrectal ultrasound (TRUS). These findings are consistent with previous studies, which have also shown the superiority of PSAD over PSA alone in detecting PCa (20). Furthermore, our study highlights the potential of PSAD as a potential screening tool for PCa, particularly in populations with a high prevalence of BPH, which can lead to elevated serum PSA levels. PSAD that can identify patients at high risk of PCa may reduce unnecessary prostate biopsies that may cause discomfort to patients. Further research, however, is needed to fully understand the potential use of PSAD in PCa screening.

Additionally, we have found that UTI was significantly higher in BPH compared to PCa. Consistency, Asafo-Adjei et al. reported 9 out of 10 urinary tract outlet obstructions resulted from prostate diseases; BPH and PCa, and the fifth of the cases were caused by BPH (21). The aging prostate tends to turn from acidic to alkaline pH in addition to, age-related low immunity that induces the predisposition to bacterial infection (22, 23). The most common type of UTI is the Gram –ve bacteria (24, 25). The mean prostate size was 69 mL in BPH cohort. Other explanations of high UTI incidence (62%) in BPH patients are increased prostate volume size, urinary retention, urethral catheterization or cystoscopy, incomplete bladder emptying, and urinary stasis (23).

According to the multivariate analysis in **Figure 1A**, UTIs in this study played a protective role in lowering the risk of PCa. UTI association with PCa is not clearly defined in the literature, studies found that urinary symptoms are not caused by PCa, even though they are positively associated with localized, but not advanced stage or life-threatening (26, 27). Similar to our findings, Collin et al., 2008 reported that UTI was associated with a higher rate of benign prostate disease than PCa in men (26, 27). However, a study by Fan et al., 2017 reported that due to tissue inflammation and the formation of free radicals, cystitis and urethritis increase PCa risk in Taiwanese men (28).

## Strength and limitations

While this study provides important insights into the risk factors and outcomes associated with PCa in Sharjah, several limitations must be considered. The study cohort was limited to a specific geographic region, and the findings may not be generalizable to other populations. Additionally, the retrospective design of our study may have introduced selection biases. For instance, some of the patients diagnosed with high-grade PCa may have been referred to the hospitals where our study was conducted, leading to the overrepresentation of high-grade cases in our cohort. Furthermore, genetic factors may play an important role in PCa risk and outcomes, which was not measured in our study.

The results of this study provide valuable information regarding the patient-specific factors associated with PCa development and mortality in Sharjah. There is a need for further studies using prospective designs to confirm these findings and to identify additional risk factors and protective factors that may influence PCa outcomes. It is important that clinicians consider these findings in the management and treatment of PCa patients, particularly when predicting patient outcomes with the Gleason score.

## Data availability statement

The data analyzed in this study is subject to the following licenses/restrictions: It is available upon request from the corresponding author. Requests to access these datasets should be directed to zalshareef@sharjah.ac.ae.

## Ethics statement

The studies involving human participants were reviewed and approved by UHS-HERC-085-10012022. The patients/participants provided their written informed consent to participate in this study.

## Author contributions

ZA conceived and designed the experiments. RA-S performed experiments. FSSA analyzed the data. All authors contributed to the article and approved the submitted version.
